# Fabrication of a Porous Fiber Cladding Material Using Microsphere Templating for Improved Response Time with Fiber Optic Sensor Arrays

**DOI:** 10.1100/2012/876106

**Published:** 2012-05-02

**Authors:** Paul E. Henning, M. Veronica Rigo, Peter Geissinger

**Affiliations:** Department of Chemistry and Biochemistry, University of Wisconsin-Milwaukee, 3210 North Cramer Street, Milwaukee, WI 53212, USA

## Abstract

A highly porous optical-fiber cladding was developed for evanescent-wave fiber sensors, which contains sensor molecules, maintains guiding conditions in the optical fiber, and is suitable for sensing in aqueous environments. To make the cladding material (a poly(ethylene) glycol diacrylate (PEGDA) polymer) highly porous, a microsphere templating strategy was employed. The resulting pore network increases transport of the target analyte to the sensor molecules located in the cladding, which improves the sensor response time. This was demonstrated using fluorescein-based pH sensor molecules, which were covalently attached to the cladding material. Scanning electron microscopy was used to examine the structure of the templated polymer and the large network of interconnected pores. Fluorescence measurements showed a tenfold improvement in the response time for the templated polymer and a reliable pH response over a pH range of five to nine with an estimated accuracy of 0.08 pH units.

## 1. Introduction

Optical fiber sensing has been applied to a large range of measurement tasks, including voltage, strain, pressure, temperature, humidity, viscosity, and chemical species [1, 2]. Fiber optic sensors are ideally suited for remote monitoring because light can be transmitted over long distances with little attenuation. Optical fibers are relatively immune to electromagnetic interference, and sensitive personnel and equipment are removed from harsh sensing environments [3]. While the most common form of fiber sensors used today functions solely at the distal end of a fiber, an evanescent-wave sensor allows for sensing over the entire length of the fiber and/or for monitoring multiple parameters simultaneously with a single fiber.

 According to Maxwell's equations, a standing wave, known as the evanescent wave, is generated outside of the fiber core when light undergoes total internal reflection at the fiber core/fiber cladding interface [4]. The evanescent wave can optically excite sensor molecules within close proximity to, but outside of, the fiber core. Similarly, fluorescence signals emitted by sensor molecules following evanescent excitation can be captured into a guided fiber mode [5]. These evanescent fields decay exponentially from the fiber core into the fiber cladding normal to the core/cladding interface. The penetration depth of the evanescent wave depends on the refractive indices of core and cladding, on the wavelength of the light, and on the incident angle of the light on the core/cladding interface. Thus, the range of the evanescent fields varies greatly for multimode optical fibers. For practical purposes, the penetration depth is on the order of the wavelength of the light. Many such sensor regions may be created along a single fiber, thus allowing for monitoring of multiple parameters simultaneously, a single parameter redundantly, or any combination thereof.

**Figure 1:**
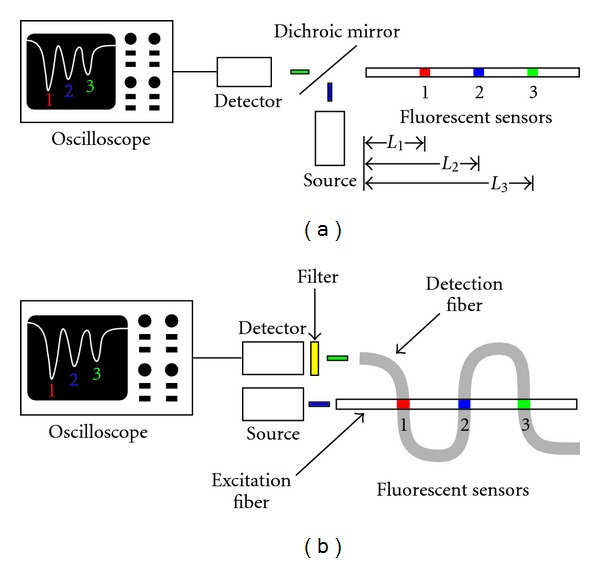
Illustration of optical time-of-flight detection (OTOFD) with (a) the single fiber scheme and (b) the crossed-fiber scheme.

Spatially resolved readout of individual sensors can be obtained by using a pulsed excitation light source and employing optical time-of-flight detection (OTOFD). In this technique, each sensor region has a unique optical pathlength from the source to the detector, and hence, the detected fluorescence pulses will have a unique and characteristic time delay corresponding to the respective pathlength [6–9]. Spatially resolved readout is obtained by spacing the sensors along the fiber so that the fluorescence pulses do not overlap. OTOFD can be implemented with one or more optical fibers, as illustrated in Figure 1. For the single fiber scheme and a modest time resolution of 25 ns, adjacent sensor regions require a 2.6-meter separation in order to resolve the fluorescence peaks appropriately [10].

Adjacent sensors can be closely spaced by employing a second optical fiber to provide the necessary time delay for full spatial resolution [10, 11], as shown in Figure 1(b). In this two-fiber scheme, the sensor regions are placed at the junctions of two crossed optical fibers, and the sensors can be spaced as closely as 1 mm [12]. One optical fiber carries the excitation light pulse and is referred to as the “excitation fiber.” After excitation of the sensor molecules through evanescent fields, their fluorescence pulses are captured by the second fiber through evanescent fields. This second fiber, referred to as the “detection fiber”, is selected such that the sensor regions have a much larger spacing (i.e., pathlength) that is sufficient to resolve the fluorescence pulses arriving at the detector. Of course, the roles of the fibers may be reversed, with the excitation fiber providing the optical time delay. Furthermore, coupling of the excitation signal to the detection fiber is low when the two optical fibers have a ninety-degree crossing angle [13], thereby increasing the signal-to-noise ratio of the fluorescence signal.

 To fabricate an evanescent-field sensor, the original fiber cladding has to be removed to expose the fiber core and, subsequently, has to be replaced with a suitable cladding that hosts the sensor molecules. This replacement cladding must meet several criteria for fiber sensing. First, the material should be optically clear in the visible range and should not increase absorption and scattering losses of light from the fiber core. Secondly, the refractive index of the cladding material should match that of the original cladding in order to maintain the original guiding conditions in the optical fibers. Thirdly, the replacement cladding needs to be rigid. Otherwise, small fiber displacements can significantly change evanescent coupling resulting in inconsistent measurements. Finally, the replacement cladding should have a rapid response time to the target analyte and exhibit limited leaching of the fluorosensor molecules. Poly(ethylene) glycol diacrylate (PEGDA) is one candidate as a replacement cladding. This polymer has high clarity in the visible range [14]. PEGDA can be cross-linked to provide a rigid material with limited swelling in water. Also, the degree of cross-linking can be controlled in order to match the refractive index of the cured polymer to the original cladding. Moreover, PEGDA is nontoxic to aqueous organisms and has a high resistance to biofouling and protein adhesion [14, 15]. The requirement for a rapid response time can be fulfilled via microsphere templating.

 In the microsphere templating technique, sacrificial microspheres are used as a template for creating a porous structure. A liquid polymer is applied to fill the voids between adjacent microspheres. After the polymer has been cured, the microspheres are either thermally removed or chemically etched to produce the skeletal structure containing many interconnected pores [16–18]. Here, microsphere templating with polystyrene microspheres was used to create highly porous crossed-fiber junctions with poly(ethylene) glycol diacrylate (PEGDA) hydrogel as a replacement cladding. In order to minimize leaching of the pH fluorosensor from the porous cladding material, fluorescein acryl amide was covalently attached to the polymer during photopolymerization [19–21]. The pore network and morphology of the replacement cladding material was examined with scanning electron microscopy (SEM). Fluorescence measurements with the crossed-fiber scheme and this porous cladding material showed a tenfold improvement of the response time resulting from the pore network and a reproducible pH response.

## 2. Experimental

### 2.1. Materials

 Dry polystyrene microspheres with a 950 nm mean diameter were purchased from Bangs Labs (Fishers, IN). Acetone, ethanol, toluene, and poly(ethylene glycol) diacrylate with a number-average molecular weight (*M*_*n*_) of 575, 2,2-dimethoxy-2-phenylacetophenone, and acryloyl chloride were purchased from Sigma-Aldrich (Milwaukee, WI). Fluorescein acryl amide was synthesized according to the literature [20] using acryloyl chloride and fluoresceinamine, Isomer I from Research Organics (Cleveland, OH). Optical fiber with a 200 *μ*m core diameter and high OH content (FT-200-UMT) was purchased from Thor Labs, Inc. (Newton, NJ).

### 2.2. Fiber Preparation

 First, to create the sensor regions, small sections of the fiber cladding were removed from a fifteen-m-long excitation fiber and a six-meter-long detection fiber. A thin sheet of aluminum with an eleven mm diameter hole was used as a mask to precisely control the length and position of the section where the cladding was removed. The optical fiber was placed on top of the metal mask, centered across the hole, and secured with cellophane tape. The buffer and cladding layers were thermally removed using a butane lighter positioned approximately six centimeters below the metal mask. The optical fiber was heated for no longer than ten seconds to prevent thermal damage to the fiber core. Next, the stripped sections were removed from the mask, cleaned with an acetone-soaked KimWipe, and allowed to dry in air. Afterwards, the fibers were mounted in shallow, orthogonal grooves machined into a polypropylene block for further work.

### 2.3. Junction Fabrication

A precursor solution of the sensor and replacement cladding was prepared with 5.0 mg of fluorescein acryl amide (pH fluorosensor), 10.0 mg of the polystyrene microspheres, 5.0 *μ*L of ethanol (to aid dissolution of the fluorosensor), 160.0 *μ*L of distilled water (to control the refractive index of the cured polymer), and 240.0 *μ*L of PEGDA-575 (polymer) containing 1% (w/v) 2,2-dimethoxy-2-phenylacetophenone (photoinitiator). The precursor solution was mixed for five minutes using a vortex mixer. A 0.5 *μ*L droplet of the precursor solution was placed on the crossed-fiber junction using an automatic pipette. The polymer was cured for thirty seconds using a PTI Xenon arc lamp operating at 75 W (Birmingham, NJ). The junction was submerged in toluene for 48 hours to remove the microspheres and then allowed to air dry for one hour. The sensor junction was stored in distilled water to prevent cracking and deformation as the coating dries.

### 2.4. Fluorescence Measurements

**Figure 2:**
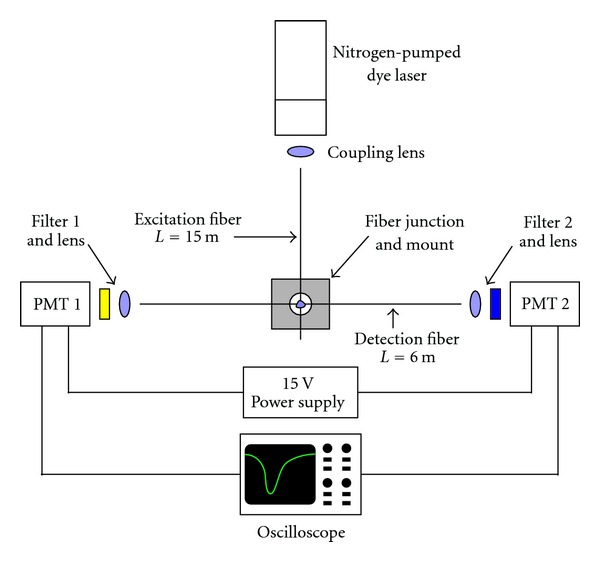
Optical setup for fluorescence measurements with a crossed-fiber pH sensor.

The optical setup is shown in Figure 2. A PTI nitrogen-pumped dye laser (Birmingham, NJ) provided 465.0 nm excitation light. Two Hamamatsu H6779-20 photomultiplier tubes (PMTs) were employed to simultaneously record the emission and excitation intensities; the excitation signal was used to compensate for source fluctuations. Each end of the detection fiber was connected to a PMT with a mount containing a collimating lens and filter from Edmund Optics (Barrington, NJ). The fluorescence signal was collected with a 515 nm longpass (OG515) filter, and the excitation signal was collected with a 467-nm interference filter with a 10 nm FWHM bandpass. The excitation and emission intensities were recorded and internally averaged with a LeCroy 1-GHz digitizing oscilloscope (Chestnut Ridge, NY). The oscilloscope averaged twenty laser pulses for pH measurements and 300 laser pulses for pH measurements. The waveforms were numerically integrated, and emission-excitation intensity ratios were calculated with a LabVIEW routine.

Equation (1) was derived from the Henderson-Hasselbalch relation for use with the emission-excitation intensity ratios

(1) $$\text{pH}=\text{p}K_{a}+\log\left(\frac{R-R_{A}}{R_{B}-R}\right).$$

*R* is calculated as the ratio of the integrated emission intensity detected at one end of the detection fiber (see Figure 2) and the integrated excitation signal intensity measured at this fiber's other end. *R*_*A*_ is the measured emission-excitation ratio for the acidic form of the fluorosensor (here the fluorescein monoanion), *R*_*B*_ is measured emission-excitation ratio for the basic form of the fluorosensor (here the fluorescein dianion), and the p*K*_*a*_ is the acid dissociation constant of the fluorosensor. Nonlinear least-squares regression was performed with the experimental data and (1) using OriginPro Version 7.0.

## 3. Results and Discussion

### 3.1. Scanning Electron Microscopy (SEM)

**Figure 3:**
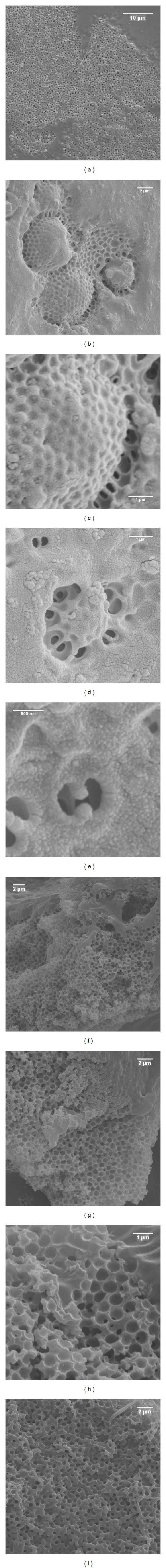
SEM images of the templated PEGDA polymer showing an interconnected pore network (a–e) on the outer surface that extends to the (f–i) inside the material. The scale bar lengths are given in parenthesis.

 Several crossed-fiber junctions were prepared with the templated PEGDA polymer for SEM imaging. Also, some samples were cleaved with a razor blade to examine the internal structure of the templated polymer. As shown in Figure 3, the microsphere templating technique created an interconnected pore network that extends throughout the replacement cladding material. Also, strong microsphere aggregation was evident in the images. This behavior is expected because the microspheres are hydrophobic and PEGDA is hydrophilic. Although some areas resemble a hexagonal close-packed structure, microsphere aggregation did not result in a highly ordered pore network. This random pore orientation is expected since no effort was made to tightly pack the microspheres. Such a dense, highly ordered arrangement is expected to be detrimental to the mechanical stability of the polymer framework after microsphere dissolution; recall that the framework structure has to lock the two fibers at the sensor junction rigidly in place. Any movement of the fibers could lead to unwanted changes in the measured sensor signal: the exponential dropoff of evanescent fields implies that small distance changes lead to exponentially amplified changes in the evanescent field strength. Ultimately, the most crucial figure of merit is the rate of analyte penetration. Even though the disordered channel structure varies between sample, their analyte penetration rates are reproducible.

On the surface of the polymer, microsphere aggregation created regions with high pore density and regions with low pore density, like that shown in Figure 3(a). Also, microsphere aggregation resulted in the three-dimensional dome structures with pores formed below the surface (Figures 3(b) and 3(c)). Figures 3(d) and 3(e) show the pillars formed where the PEGDA polymer filled the void between three aggregated microspheres. These images also show pore formation below the surface of the polymer coating.

 The cross-sectional images from the cleaved junctions, Figures 3(f) through 3(h), show that pores were formed throughout the structure. In addition, these images show a higher degree of microsphere aggregation resulting in a denser pore network, resembling a sponge. This pore network should accelerate diffusion of an aqueous analyte through the PEGDA-based replacement cladding to the sensor molecules located near the fiber core and therefore yielding an improved response time of the sensor.

### 3.2. Response Time

 Response times were measured to determine if the pore network accelerated transport of an aqueous analyte through the polymer replacement cladding. A non-templated sensor junction was created to provide a reference time value for comparison. The response times were measured by submerging the sensor junctions into phosphate buffer solutions under static equilibrium (i.e., no stirring). The fluorosensor has a pH-dependent response over a pH range from four to ten, resulting from an increase in both absorbance and fluorescence quantum yield when the pH is increased.

**Figure 4:**
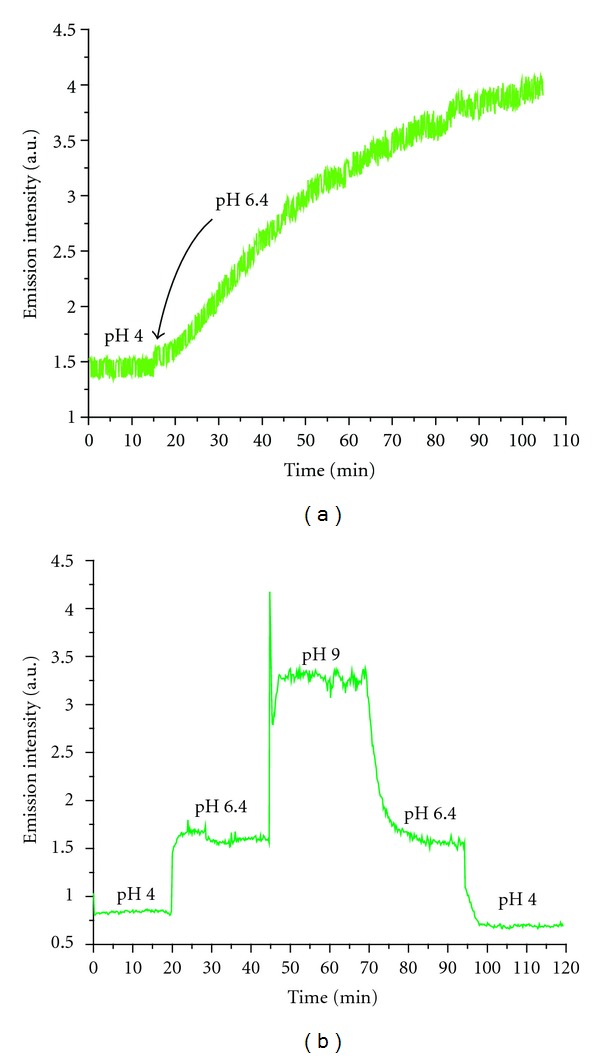
Fluorescence curves of the (a) non-templated and (b) templated sensor junctions showing a tenfold improvement of the response time. Each datapoint represents the average of twenty laser excitation pulses.

The response-time traces shown in Figure 4 demonstrate that microsphere templating improves response time significantly. For the nontemplated sensor junction, the change in fluorescence was gradual and the time to reach equilibration was in excess of 100 minutes. Conversely, the templated sensor junction had a rapid initial response followed by an equilibration time of ten minutes. Thus, the pores formed by microsphere templating increase diffusion of the analyte to the sensor molecules near the fiber core, yielding a tenfold improvement in the sensor response time.

As the pore network increases diffusion of the analyte throughout the replacement cladding material, increased leaching of the sensor is expected. Leaching can be minimized by covalently attaching the sensor dye to the polymer matrix. Here, the pH sensor was covalently tethered to PEGDA during photopolymerization.

### 3.3. pH Measurements

**Figure 5:**
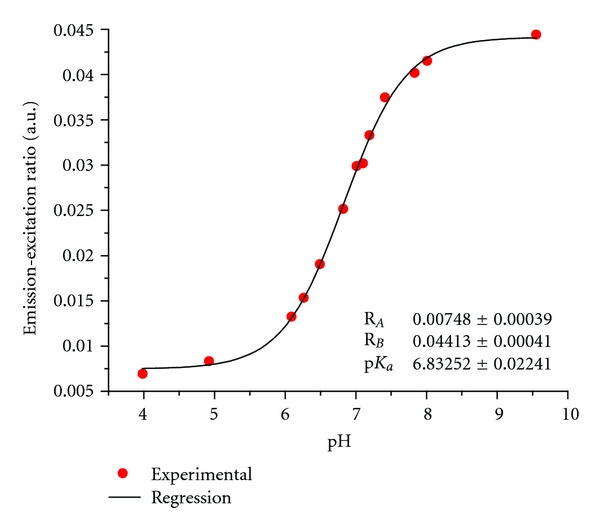
pH response from a crossed-fiber sensor junction using integrated emission-excitation ratios. Regression was performed with (1). Each experimental datapoint represents the average 300 laser excitation pulses.

Fluorescence-based pH measurements were taken while the sensor junction was submerged in different phosphate buffer solutions. Both the excitation and emission intensities were recorded and the emission-excitation ratios were determined. The resulting pH response is shown in Figure 5.

The pH response of the crossed-fiber sensor is consistent with the pH-dependent emission of fluorescein. The p*K*_*a*_ value of the fluorosensor was determined to be 6.8 from regression analysis. This value corresponds well to fluorescence-lifetime-based pH measurements taken in our lab with the same crossed-fiber pH sensor [22]. The p*K*_*a*_ values of the sensor dye reported in the literature vary for different host polymer matrices. The observed p*K*_*a*_ of fluorescein acryl amide was determined to be 6.1 in solution (i.e., unbound) and 7.5 when covalently attached to a poly(hydroxyethyl methacrylate) matrix [20]. Elsewhere, the observed p*K*_*a*_ of the fluorosensor in a polyacrylamide polymer was 6.5 [19]. Finally, a p*K*_*a*_ value of 6.6 was determined for this sensor using poly(hydroxyethyl methacrylate) cross-linked with PEGDA [21]. The pH response and the p*K*_*a*_ value of 6.8 determined here are consistent with other published values.

The accuracy of the sensor system was estimated from the pH deviations with respect to a validation method. First, pH of the phosphate buffer solutions was measured with a conventional pH electrode with a specified accuracy of 0.05 pH units. The fluorescence of the sensor junction was recorded using these phosphate buffer solutions. The pH deviations were determined from the pH electrode measurements and the pH calculated from the fluorescence data and the regression line. The largest pH deviation was 0.08 pH units within ±1 pH units of the p*K*_*a*_, and a majority of the deviations here were 0.02 pH units over the useful sensing range of the fluorosensor. Thus, the accuracy of the sensor system is estimated to be 0.08 pH units or better.

 The porous fiber sensor cladding yielded a response that is fast, reversible, and does not exhibit significant hysteresis or “memory effects.” Although PEGDA was selected for sensing in aqueous environments, the microsphere templating technique can be applied with other polymers for different sensing requirements and thus has potential for many applications. Moreover, the rigid cladding scaffold locks the two-fibers in a sensor junction in place, eliminating response-time and swelling issues observed with nontemplated, hydrophilic cladding materials. Moreover, an array of crossed-fiber junctions can be made for multi-analyte sensing. Also, as both ends of the detection fiber can be used, ratiometric techniques can be readily employed using dual excitation and dual-emission fluorophores like carboxynaphthofluorescein [23] and seminaphthorhodamine [24], or a combination of two fluorophores where one dye serves as reference [25, 26].

## 4. Conclusions

A replacement fiber optic cladding was made with a cross-linked poly(ethylene) glycol diacrylate (PEGDA) polymer. This polymer was made porous through the microsphere templating technique. Scanning electron microscopy verified that the microsphere templating technique created an interconnected pore network throughout the cladding material. Fluorescence measurements fluorescein-based pH sensor showed that the microsphere-templated polymer had a tenfold improvement in the response time resulting from increased diffusion of the target analyte through the replacement cladding containing sensor molecules. Also, fluorescence measurements demonstrated that the replacement cladding provided a reliable response with an estimated accuracy of 0.08 pH units. The microsphere templating technique can potentially be applied to a wide range of sensor materials including fiber optic sensor systems.
